# Identification of new, well-populated amino-acid sidechain rotamers involving hydroxyl-hydrogen atoms and sulfhydryl-hydrogen atoms

**DOI:** 10.1186/1472-6807-8-41

**Published:** 2008-10-07

**Authors:** Bosco K Ho, David A Agard

**Affiliations:** 1 Department of Biochemistry, Howard Hughes Medical Institute, University of California, San Francisco, San Francisco CA 94158-2517

## Abstract

**Background:**

An important element in homology modeling is the use of rotamers to parameterize the sidechain conformation. Despite the many libraries of sidechain rotamers that have been developed, a number of rotamers have been overlooked, due to the fact that they involve hydrogen atoms.

**Results:**

We identify new, well-populated rotamers that involve the hydroxyl-hydrogen atoms of Ser, Thr and Tyr, and the sulfhydryl-hydrogen atom of Cys, using high-resolution crystal structures (<1.2 Å). Although there were refinement artifacts in these structures, comparison with the electron-density maps allowed the placement of hydrogen atoms involved in hydrogen bonds. The χ2 rotamers in Ser, Thr and Cys are consistent with tetrahedral bonding, while the χ3 rotamers in Tyr are consistent with trigonal-planar bonding. Similar rotamers are found in hydrogen atoms that were computationally placed with the Reduce program from the Richardson lab.

**Conclusion:**

Knowledge of these new rotamers will improve the evaluation of hydrogen-bonding networks in protein structures.

## Background

One important conformational parameter of a protein structure is the sidechain χ torsion angle [1]. In crystal structures, these torsion angles were found to be rotameric [2]: they cluster around specific values, values that can be explained in terms of relatively simple stereo-chemical considerations [3,4]. Consequently, libraries of sidechain rotamers have been compiled [5,6]. These libraries have proven useful in parameterizing sidechain conformations for homology modeling [7], monte-carlo simulations [8], and protein design [9]. Rotamer libraries are also used to build and verify crystallographic models [10]. Although the sidechain rotamers have been extensively studied, there remain a number of rotamers involving hydrogen atoms that have been overlooked.

Due to the difficulty in placing hydrogen atoms in protein electron density maps, it has long been customary to omit hydrogen atoms in reporting the crystal structure of a protein. However, Richardson and co-workers showed that positions of hydrogen atoms in high-resolution crystal structures can be confidently projected from the topology of the heavy atoms [11]. The projected hydrogen atoms, in most cases, form better van-der-Waals contacts with the neighboring atoms than do the heavy atoms themselves. The heavy atoms accommodate the packing of the hydrogen atoms, even though the hydrogen atoms cannot be seen in the crystal structure.

One of the reasons why the projection of hydrogen atoms works so well is that the positions of most of the sidechain hydrogen atoms are stereo-chemically restricted. For example, the Hβ atom of Val can only adopt one tetrahedral-bonding position off the Cβ position given that 3 other C atoms are also bound to Cβ. For other hydrogen atoms, symmetry between equivalent methyl-hydrogen atoms results in similar restrictions. For example, in Val, the three equivalent Hγ1 atoms bound to Cγ1 saturate the three available tetrahedral-bonding positions at Cγ1. Nevertheless, there exist four types of sidechain hydrogen atoms in which there is ambiguity in projecting their positions. For instance, in Ser, there are three different ways to place the Hγ atom onto the tetrahedral-bonding positions of the Oγ atom. This freedom is also found in the hydroxyl-hydrogen atoms of Thr and Tyr, and in the sulfhydryl-hydrogen in Cys.

As the positions of most of the sidechain hydrogen atoms are so restricted, little attention has been paid to their conformation in crystal structures. However, given the growing number of structures containing hydrogen atoms in the data bank, it has become practical to revisit the question of sidechain hydrogen rotamers for the four classes of ambiguous sidechain hydrogen atoms. The positions of these sidechain hydrogen atoms should be parameterized by χ torsion angles, and we would like to know if these angles display rotameric preferences. Here, we study the distributions of these χ torsion angles in three data-sets: (1) high-resolution X-ray structures that contains explicit hydrogen atoms, (2) neutron diffraction structures and (3) structures with computationally-placed hydrogen positions.

## Results

### The rotamers in high-resolution X-ray structures with hydrogen atoms

For the first part of the analysis, we use high-resolution X-ray structures that have explicit hydrogen atoms in the Ser, Thr, Tyr and Cys residues. As such, the data-set consists of structures found in the RCSB.ORG website [12] with resolution < 1.2 Å, where hydrogen atoms are found in the structure. The hydrogen atoms are filtered for residues with no alternate conformations and where the neighboring heavy atom has a B-factor < 40. The structures were further selected depending on the availability of the electron density maps in the Electron Density Server [13]. This results in 27 structures: 1AHO, 1DY5, 1JM1, 1M40, 1RW1, 2AXW, 2FDN, 1BXO, 1EUW, 1KQP, 1MUW, 1TT8, 2BF9, 2FFY, 1C75, 1F94, 1L9L, 1O7J, 1UCS, 2CAL, 2H5C, 1CEX, 1GQV, 1LS1, 1RB9, 2AWK, 2ERL.

**Figure 1:**
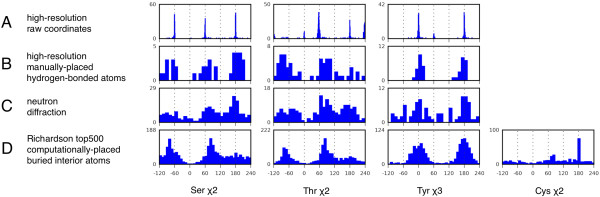
**Distributions of the hydrogen-based χ angles of Ser, Thr, Tyr, and Cys.** (A) The distributions calculated directly from high-resolution (<1.2 Å) structures deposited in the PDB. (B) The distributions of the same structures where the hydrogen atoms have been manually placed to fit the electron-density of hydrogen-bonded hydrogen atoms. (C) The distributions from the structures determined by neutron diffraction. (D) The distributions from the hydrogen atoms that have been computationally-placed with Reduce from the top500 database of non-redundant structures from the Richardson lab.

We first generate the χ-angle distributions of the hydrogen atom positions directly from the coordinates reported in the high-resolution structures (Figure 1A). Hydrogen atoms were found for 232 Ser, 356 Thr and 187 Tyr. The distributions show exceptionally sharp peaks, which correspond to rotameric preferences. In Ser, the hydrogen atom position is defined by χ2 = Cα-Cβ-Oγ-Hγ, where the χ2 distribution shows three peaks, corresponding to tetrahedral bonding on the Oγ atom. In Thr, the hydrogen atom position is defined by χ = Cα-Cβ-Oγ1-Hγ1, where the distribution shows 4 peaks. Two of the peaks are expected from tetrahedral bonding but the peak χ2 = -60° is missing. Instead there are two other peaks at χ2 = -120° and χ2 = 0°. In Tyr, the hydrogen position is defined by χ3 = Cδ1-Cε1-Oζ-Hζ where the distribution has three peaks, of which 2 are expected from trigonal-planar bonding and there is an unexpected peak at χ3 = 60°.

**Figure 2:**
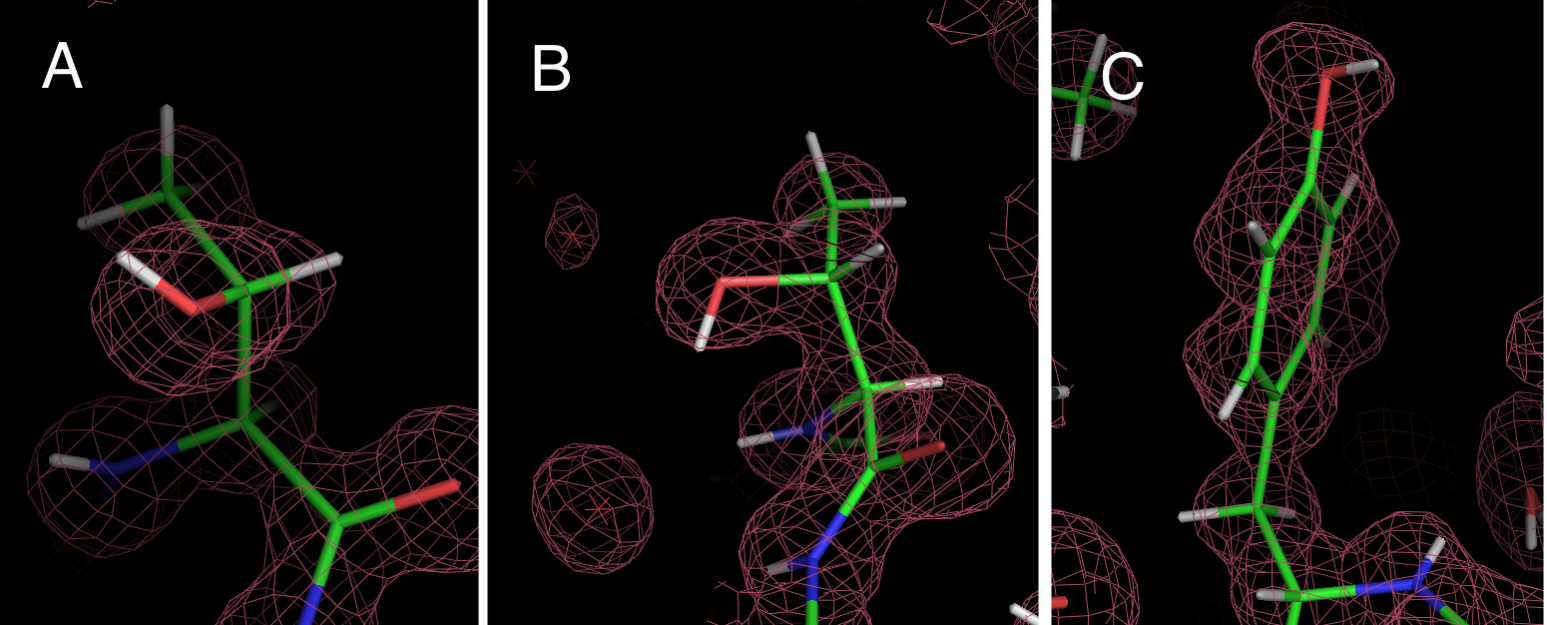
**Examples of the poor fits of the hydrogen positions to the 2F_0_-F_c_electron-density map (at 0.5σ contour level) in high-resolution X-ray structures.** These conformations are refinement artifacts (A) Thr-A41 [1C75] at χ2 = -120°, (B) Thr-A104 [1JM1] at χ2 = 0°; and (C) Tyr-A3 [1RW1] at χ3 = 60°.

In many cases with high-resolution structures, the hydrogen atoms are not actually refined, but are instead placed automatically. As such their positions can be unreliable. To check this, we carefully inspected the 2F_0_-F_c _maps for the hydrogen atoms of the 27 structures in this data-set and discovered that no electron density was observable for any of these hydrogen atoms at normal contour levels of the maps (Figure 2). Therefore, the reported positions of these hydrogen atoms must be an artifact of automatic placement. The sharp peaks in Figure 1 arise from pre-defined restrictions on the χ angles, and not intrinsic structural propensities. Of the 27 structures, there was one notable exception in 2H5C, which contains a large number of rotamers found far from the pre-defined rotamers (> 10°) where the hydrogen atom positions have been carefully refined against the chemistry of nearby atoms.

Given the absence of agreement with the other distributions (below), and incompatibilities with tetrahedral-bonding and trigonal-planar bonding, it must be concluded that in Ser, the two rotamers at χ2 = -120° (Figure 2A) and χ2 = 0° (Figure 2B) are artifacts especially since these rotamers pack the hydrogen atom against a carbon atom. Similarly, in Tyr, the off-planar rotamer at χ3 = 60° (Figure 2C) is an artifact.

### Manually-placed rotamers in X-ray structures using hydrogen-bonds

**Figure 3:**
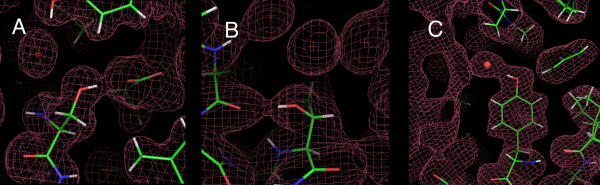
**Examples of hydrogen positions that were manually-placed to the 2F_0_-F_c_electron-density of a hydrogen-bond (at the 0σ contour level).** (A) Ser-A205 [2AWK] with a hydrogen bond to a Glu. (B) Thr-A30 [1LS1] with a hydrogen bond to a backbone carbonyl O atom. (C) Tyr-149 [1CEX] with a hydrogen bond to water.

In general, the 2F_0_-F_c _electron density for the hydroxyl-hydrogen atoms is too weak to be observed at contour levels that are typically used. However, in these high-resolution structures, even at a contour level of σ = 0, there are regions of the 2F_0_-F_c _map that result in well-defined contours of the molecule. Given that heavy atoms can be clearly distinguished in these maps, in regions where the map is well defined, the position of hydrogen atoms can be determined from density between the donor and acceptor atoms of a hydrogen bond (Figure 3). Such hydrogen-bonded hydrogen atoms were identified for 37 Ser, 39 Tyr and 60 Thr residues, and the hydrogen atom positions were manually placed [see Additional File 1].

The χ-angle distributions and rotamers were calculated from these manually-placed hydrogen atoms (Figure 1B and Table 1; also see Additional File 2). The Ser χ2 distribution splits into three rotamers at -79°, 69° and 189° (Figure 1), corresponding to the three angles of tetrahedral-bonding of the Hγ atom to the Oγ atom. The 189° rotamer is favored, presumably because it positions the Hγ atom furthest away from the Cβ atom. The Thr χ2 distributions contain two well-populated rotamers at χ2 = -74° and 80°. Surprisingly, there is no clear peak at the tetrahedral-bonding position that places the hydrogen atom away from the bulk of the sidechain. The Tyr χ3 distribution splits into two rotamers at 5° and 174°, consistent with trigonal-planar bonding (Figure 1). However, given that the aromatic ring is symmetric, these two rotamers are equivalent.

**Table 1:** Rotamers from hydrogen-bonded hydrogen atoms fitted from electron density in high-resolution structures.

**Type of χ Angle**	**Rotamer**	**Population**
		

Ser χ2: 37 counts	-79 ± 18°	27%

	69 ± 24°	30%

	189 ± 16°	43%

		

Thr χ2: 60 counts	-74 ± 26°	47%

	80 ± 18°	35%

	176 ± 35°	18%

		

Tyr χ3: 39 counts	5 ± 9°	49%

	174 ± 12°	51%

### Comparison with neutron structures

We can compare the high-resolution manually-placed distributions to distributions from neutron structures. While the neutron structures are generally of a lower resolution, hydrogen is a strong and negative neutron scatterer, which should allow reasonably accurate positioning of the hydrogen atoms. We found 16 neutron structures in the PDB with hydrogen atoms, containing a total of 215 Ser, 154 Thr and 85 Tyr residues. There were no Cys hydrogen atoms. In keeping with the lower resolution, the distributions in the neutron structures (Figure 1C) are more diffuse than those of the high-resolution X-ray structures (Figure 1B). In the neutron structures, the Ser distribution has a peak near 180°. In Thr, the distribution has one delineated peak at 60°. In Tyr, the distribution is diffuse with weak peaks at 0° and 180°. Qualitatively, there is agreement with distributions derived from the manually-placed hydrogen atoms of the high-resolution crystal structures (Figure 1B).

### Comparison with computationally-placed hydrogen atoms

We can also compare the high-resolution manually-placed distributions to distributions derived from computationally-placed hydrogen atoms. This allows us to evaluate algorithms that project hydrogen positions from the coordinates of heavy atoms. We use a representative non-homologous set of high resolution structures (< 1.8 Å), provided by the Richardson lab [6], where missing hydrogen atoms have been computationally-placed using the program Reduce [14]. These hydrogen atoms were computationally placed by optimizing hydrogen bonds and steric contacts with neighboring atoms.

In the structures with Reduce-placed hydrogen atoms, we found an artifact in the surface hydrogen atoms. As there are few neighboring contacts on the surface to help determine the position of hydrogen atoms, many surface hydrogen atoms remain at the default value, resulting in a pronounced peak at 180° (data not shown). This peak can be removed if we eliminate surface residues. Furthermore, as the Reduce algorithm uses steric contacts to optimize hydrogen positions, we need to use well-packed hydrogen atoms. Consequently, we only consider buried interior hydrogen atoms, defined as atoms with > 8 neighboring atoms, where a neighboring atom is defined if it is within 3.5 Å of another atom. We also filter out residues with alternate conformations and atoms where the B-factor > 40. There were hydrogen atoms from 5768 Ser, 5932 Thr, 3645 Tyr and 660 Cys residues in 480 structures. The large size of this data set gives the most reliable statistics.

The χ-angle distributions and rotamers were calculated from the Reduce-placed hydrogen atoms (Figure 1D and Table 2; also see Additional file 2). In the χ2 distribution of Ser, there are only two peaks, as opposed to three peaks in the high-resolution crystal structures (Figure 1). Both the Thr and Tyr distributions qualitatively reproduced the manually-placed hydrogen atom distributions. Apart from the missing χ2 = 189° rotamer in Ser, the Reduce-placed rotamers show remarkable agreement with the manually-placed rotamers. One possible reason for this agreement is that Reduce algorithm makes extensive use hydrogen-bonding optimization, which reflects the choice of using hydrogen-bonding density to manually place the hydrogen atoms in the high-resolution structures.

**Table 2:** Rotamers from computationally-placed hydrogen atoms in the Richardson set of structures.

**Type of χ Angle**	**Rotamer**	**Population**
		

Ser χ2: 5768 counts	-75 ± 25°	30%

	81 ± 22°	30%

	180 ± 26°	40%

		

Thr χ2: 5932 counts	-70 ± 24°	25%

	80 ± 20°	37%

	178 ± 24°	38%

		

Tyr χ3: 3645 counts	2 ± 27°	41%

	180 ± 23°	59%

		

Cys χ2: 660 counts	-66 ± 32°	20%

	72 ± 27°	38%

	179 ± 24°	42%

Given the robust performance of Reduce, we investigated the position of sulfhydryl-hydrogen atoms in the set of structures provided by the Richardson lab. The hydrogen atoms are considered only if there are no other Cys residues within 4.5 Å of the SG atom of the Cys in order to avoid disulfide-bonded Cystines. The position of the sulfhydryl-hydrogen in Cys is determined by the χ2 = Cα-Cβ-Sγ-Hγ angle. The Cys χ2 distribution show a dominant rotamer at χ2 = 181° (Figure 1), which places the hydrogen atom furthest away from the backbone, in between the two Hβ atoms.

## Methods

Data was collected from PDB structures using in-house Python scripts. The distributions in Figure 1 were binned using a rough guideline of ~4 bins for a peak. For the X-ray structures, 360 bins were used for the distribution from the raw coordinates, and 30 bins for the distributions from the manually-placed hydrogen atoms. For neutron structures, we used 30 bins. For the computationally-placed distributions, we used 50 bins. To calculate the means and distributions for the rotamers, we divided up the χ range into 3 equal partitions in Ser, Thr and Cys, and 2 partitions for Tyr.

## Conclusion

Based on experimental data, we find that certain sidechain torsion angles involving hydrogen atoms have strongly preferred orientations and should thus be considered rotameric. Although there were serious artifacts found in the reported coordinates of high-resolution X-ray structures, reliable hydrogen atom positions could be directly derived from the electron-density maps of hydrogen-bonded hydrogen atoms. The χ-angle distributions of these hydrogen-bonded hydrogen atoms match the distribution of hydrogen atoms that were computationally placed by the program Reduce [14].

## Authors' contributions

BKH carried out the study and drafted the manuscript. DAA provided support and guidance. This work was supported by the Howard Hughes Medical Institute.

## Supplementary Material

Additional file 1 Manually-fitted hydrogen atoms in high resolution structures. In these high-resolution X-ray structures, the hydroxyl hydrogen atoms were removed if there is no corresponding electron density but if there is sufficient electron density due to hydrogen-bonding, the hydrogen atom position were fitted manually. Click here for file

Additional file 2 List of hydroxyl-hydrogen χ rotamers in high-resolution PDB files. List of the residues used to calculate the hydroxyl-hydrogen χ-angle rotamers in Ser, Thr and Tyr. Data-sets provided for both the manually-placed hydrogen atoms in the high-resolution structures, and the Reduce-placed hydrogen atoms in the Richardson data-set. Click here for file
